# Does Peritraumatic Ketamine Reduce Symptoms of Post-Traumatic Stress Disorder?

**DOI:** 10.51894/001c.17647

**Published:** 2020-10-30

**Authors:** Jack Brodeur, Ryley Mancine, Alyse Ley, Jed Magen

**Affiliations:** 1 Michigan State University College of Osteopathic Medicine; 2 Psychiatry Michigan State University

**Keywords:** ketamine, ptsd, prehospital, peritraumatic, propofol, asd, emegency

## Abstract

**INTRODUCTION:**

Acute stress disorder (ASD) and post-traumatic stress disorder (PTSD) are chronic diseases which can affect patients following a severe trauma. As these patients typically first present to the emergency department, it is critical for emergency physicians to remain updated on the use of ketamine or other anesthetic agents which may impede development or reduce symptoms which may impair normal functioning. The purpose of this clinical review was is to review the literature regarding how the use of peritraumatic ketamine could decrease the incidence of ASD and PTSD. In 2019, the authors completed a MEDLINE search was performed yielding 25 articles which were initially evaluated by the first and second authors. Four articles which met inclusion criteria are discussed in this manuscript.

**SUMMARY of EVIDENCE:**

Although two earlier research groups have found that peritraumatic ketamine administration contributed to increased symptoms of PTSD (e g., reexperiencing, dissociation, avoidance, and hyperarousal), two later studies have indicated that ketamine had no effect on PTSD development. Additionally, one 2012 study group has suggested propofol use may alleviate PTSD symptoms at six months post-trauma. Another 2017 study team found that the number of surgical procedures was directly correlated with increased PTSD development.

**CONCLUSIONS:**

Based on the literature to date, peritraumatic ketamine does not appear to influence the prevention nor development of ASD and subsequent PTSD. More research is needed to clarify the psychopharmacologic effects of ketamine when used in the management of reactions to acute trauma experiences. Based on the results of the two later works, future research is indicated considering whether propofol may contribute to PTSD development.

## INTRODUCTION

Post-Traumatic Stress Disorder (PTSD) and its acute variant Acute Stress Disorder, (ASD) are unique psychiatric diagnoses with a defined cause. The onset of these trauma related disorders is often documented in the patient chart at the time or shortly after the precipitating trauma occurs.^1,2^ Patients with these diagnoses typically experience intrusive thoughts, unwanted memories, and dissociative “flashbacks” along with negative mood and hyper-arousal.^1^

These symptoms and disorders are often frustrating, frightening, and difficult for individuals who suffer from them. Duration of the disease is key for differentiating an ASD diagnosis from PTSD. If symptoms are present for under one month the disease is termed ASD, but persistence of symptoms for more than a month prompts reclassification of ASD to PTSD.^1^

Both ASD and PTSD are seen more frequently in women, who are more often subjected to different kinds of domestic trauma (e g., sexual assault) or are more often victims of relationship violence. PTSD is frequently diagnosed as a failure to return to homeostasis following a traumatic incident.^2^

Current treatment guidelines recommend usage of Cognitive Behavioral Therapy (CBT) or Exposure Therapy (ET) in conjunction with Selective Serotonin Reuptake Inhibitors (SSRI) (e.g., Sertraline/Zoloft or Fluoxetine/Prozac) for the treatment of PTSD in clinic-based settings after safety and crisis intervention measures have taken place. ^1^ Although ASD is similarly treated, SSRIs are often not prescribed immediately to evaluate the effectiveness of a purely CBT/ET-based treatment.

Emergency medicine physicians and advanced practice providers are uniquely posed to encounter patients who have immediately experienced a trauma and may be at risk for the future development of ASD or PTSD. Medical intervention in the emergency department (ED) immediately following a trauma may help prevent the onset of these conditions.^2^

Ketamine is an NMDA receptor antagonist with predictable pharmacodynamics that has been used in a dose-dependent fashion to attain analgesia, amnesia, akinesia, and anesthesia.^2^ These properties, along with its favorable safety profile in both adult and pediatric patients make it a versatile medication for pre-hospital and ED providers who treat acutely injured patients.^3,4^

Ketamine is also a strong antidepressant with long acting effects; a single, sub-analgesic dose of ketamine can serve to alleviate the acute symptoms of major depressive disorder for up two weeks.^5^ Additionally, recent data suggests that ketamine can alleviate PTSD symptoms in patients who have failed conventional pharmacotherapy.^6^

During recent decades, ketamine has been considered a possible prophylactic agent to prevent the emergence of PTSD-like symptoms.^1,6^ A seminal article by McGhee et al. published in 2008 discussed the use of perioperative ketamine in burned service members and reported that patients who received ketamine (n = 119) had a lower prevalence of PTSD than those who had not (n = 28).^7^

The soldiers receiving ketamine in this study demonstrated lower scores on the PTSD Checklist-Military (PCL-M)^8,9^ even though they had higher injury severity scores, received more surgical procedures, and spent more days in the intensive care unit (ICU).^7^ In a follow-up 2014 study by the McGhee group, however, ketamine was not found to lower the prevalence or incidence of PTSD.^10^

In summary, the clinical efficacy of ketamine in the acute-care setting to prevent ASD and PTSD remains largely unknown.

### Purpose of Clinical Review

This clinical review was conducted to review the available literature focusing on the use of ketamine in adults in ED or acute-care setting following a recent trauma. In 2019, the authors conducted a MEDLINE search using the search terms: ("Ketamine"[Mesh] or "ketamine" or "Ketamine"[nm] or "Ketamine/therapeutic use") and ("Stress Disorders, Post-Traumatic"[Mesh] or "PTSD") and ("Trauma" OR "War" or "Soldier" or "Motor Vehicle Accident"). This search generated a total list of 25 articles which was reviewed by the first and second authors (JB and RM).

Articles that did not pertain to ED or prehospital trauma care settings or non-traumatic ASD and PTSD patient scenarios were eliminated. Articles pertaining to ASD, PTSD, and PTSD symptoms were included. Regarding this topic, only four articles that met the authors’ inclusion criteria were identified.

## SUMMARY OF THE EVIDENCE

### Schönenberg et al. (2005)^11^

This retrospective study evaluated the effects of prehospital ketamine administration on the later development of ASD and PTSD in a sample of 56 moderately injured accident victims in Germany. Patients included in this study ranged from 17 and 66 years old (41 +/- 13.9) and had initial Glasgow Coma Scale (GCS) scores > 14 (possible range from 3 to 15).^12^ These authors had excluded patients who had sustained brain or head trauma, had pre-existing psychiatric illnesses including substance abuse, and had an Injury Severity Score (ISS) ^13^ > 16 as defined by the German Society for Trauma Surgery (i.e., on possible range from 0 to 75).

The patients in this study completed the validated German translations of the Acute Stress Disorder Scale (ASDS), the modified Peritraumatic Dissociative Experiences Questionnaire (PDEQ), and the Impact of Event Scale-Revised (IES-R).^14–16^ Participants also finished a modified version of the Traumatic Life Events Questionnaire (TLEQ) to screen for other traumatic experiences.^17^

A total of 48 male patients and eight female patients again participated approximately 12 months after sustaining their traumatic injuries (e.g., burns, contusions, fractures, cuts). These injuries had been sustained in household, recreational, or work-related accidents. Each patient had been accompanied by an ED physician during ambulance transport and received a fractionated or single dose of racemic ketamine (i.e., an equal mixture of ketamine enantiomers), (S)-ketamine, or an opioid analgesic en route to a trauma center.

Ketamine was not administered after patients reached a trauma center. A combination of fentanyl and midazolam was used for most surgical cases. Patients were given piritramide or tramadol for treatment of moderate to severe pain. The authors also noted that three patients who received opioid analgesia also received beta blockers for cardiac conditions. Additionally, three subjects who had received ketamine and eight that received opioids received one to two doses of benzodiazepines (e g., flunitrazepam, oxazepam, or lormetazepam) for sleeplessness during their hospitalization.

In summary, patients who received (S)-ketamine or racemic ketamine had significantly higher, more symptomatic PDEQ scores relative to those who received opioid analgesics, with (S)-ketamine having a significantly stronger effect than racemic ketamine (p = 0.000 and p = 0.043, respectively).^11^ (S)-ketamine also led to higher scores for dissociation (p = 0.005), reexperiencing (p = 0.027), and avoidance (p = 0.017) on the ASDS when compared to the opioid group. The (S)-ketamine group also exhibited significantly higher avoidance scores on the ASDS (p = 0.018), and higher reexperiencing and avoidance scores on the IES-R when compared to the racemic ketamine and opioid groups (p < 0.034 in all groups).^11^

Finally, (S)-ketamine significantly increased hyperarousal scores on the IES-R when compared to opioids (p = 0.006), but not racemic (i.e., single or fractionated dose) ketamine. There was no significant difference in the number or type of surgical cases between the (S)-ketamine, racemic ketamine, and opioid subgroups.

The results of several studies have suggested that (S)-ketamine is associated with fewer neuropsychological side effects than racemic ketamine.^18,19^ The results of Schönenberg et al.’s (2005) study directly conflicts with the subsequent findings concerning the neuropsychological effects of (S)-ketamine. We suspect that this conflict may be attributed to the presence of confounding variables within the study.

### Schönenberg et al. (2008)^20^

This was a follow-up study by the same group of researchers as the previously discussed 2005 study. The authors’ primary goal was to determine whether ketamine administration during medical treatment of mild to moderate traumatic injuries had an impact on the development of ASD. Subject demographics and observed exclusion criteria were similar to the 2005 design. However, two notable changes in the inclusion criteria included raising minimum the GCS score from GCS > 14 to GCS = 15 and allowing the inclusion of ED patients who received analgesic/anesthetic drugs. We suspect that the authors slightly raised the minimum GCS score to 15 to eliminate patients who had even mild cognitive impairment secondary to their traumatic brain injuries.^12^

Enrolled subjects had been administered analgesic and/or sedative drugs either in transport to or at a single trauma center ED. They were assigned into one of three study arms based upon which medication they received: racemic ketamine (n = 13), opioid analgesics (e.g., tramadol or piritramide) (n = 24), or weak Grade 1-2 non-opioid analgesics (e.g., paracetamol, metamizole, or diclofenac) (n = 13).^20^

During this study, midazolam was often combined with ketamine treatment. Each patient completed German-translated versions of the ASDS and PDEQ on the third day following their presentation to the trauma center.^14,15^ As in the 2005 study, participants took the TLEQ to identify and control for any previous traumatic experiences.^17^

Some participants underwent minor surgical procedures (e g., fracture reduction) or received additional analgesics while they were hospitalized, though there was no difference in frequency or type of these treatments or other medications (e.g., beta-blockers or benzodiazepines) reported across the three subgroups.^20^

The results of statistical analyses showed that racemic ketamine significantly increased the ASDS scores relative to opioids for the reexperiencing (*p* = 0.010), avoidance (*p* = 0.009), and hyperarousal (*p* = 0.039). Although post hoc analyses supported the initial findings that racemic ketamine led to significantly increased reexperiencing (p = 0.010) and avoidance (p = 0.009) ASDS scores, the study team concluded racemic ketamine’s effect on hyperarousal to be insignificant (*p* = 0.232).^20^

Post hoc analyses also determined that racemic ketamine administration was significantly related to increased dissociation on ASDS compared to opioids. There was no statistically significant differences found in PDEQ or ASDS scores between the opioid group and the non-opioid analgesic group.

### Usuki et al. (2012)^21^

This was a single-center prospective cohort study of motor vehicle accident victims (n = 300) conducted in Japan to examine the impact of propofol administration on the development of PTSD. Study subjects had sustained massive trauma requiring admission to the ICU and some also received ketamine within 72 hours of injury.^20^ Although this study primarily assessed the effects of propofol (i.e., a commonly used IV anesthetic, this article is significant to our discussion for two reasons.^22^

First, the authors collected data on how many patients received ketamine (n = 39). Of those patients who received ketamine, nine (23%) were in the propofol group and 30 (77%) were in the non-propofol group. Multivariate analyses demonstrated that ketamine administration did not have a significant impact on full or partial PTSD development at one month (*p* = 0.262) or at six months (*p* = 0.878) post-injury.^21^

Second, the group’s multivariate analyses indicated that propofol had significantly influenced the development of full and partial PTSD at six months post-injury (*p* = 0.009). Although the number of patients who received propofol was quite low (n = 26), the authors argued that it may be worth considering propofol to inhibit development of PTSD in longer-affected patients.

### Mion et al. (2017)^23^

This was a retrospective study involving 274 French soldiers who had been wounded in the Afghanistan war. The authors collected data from the French Military Health Services concerning: mechanisms of injury, ISS scores, head injury specifics, initial GCS scores, traumatic amputations, administration of ketamine, morphine, or midazolam on the battlefield and in field hospitals, total number of surgeries, and development of ASD.^23^ Explosive blasts were the primary mechanism of injury (n = 189, 69.0%). The remainder of the soldiers in this study were injured by firearms or in military vehicle transport accidents.

During this study, forty-nine (18.0%) soldiers were diagnosed with ASD and 98 (36.0%) with PTSD. Although 54 of 89 (61%) of soldiers who had received ketamine on the battlefield or in a field hospital developed PTSD, a logistic regression model (using data from soldiers who received either ketamine or morphine to prevent collinearity) demonstrated that ketamine was not significantly related to the development of PTSD. (*p* = 0.080)

The authors found that the number of surgical procedures and the presence of ASD were the only statistically significant covariates related to subsequent PTSD development (both factors p < 0.001). Furthermore, they used data from 50 soldiers who had received ketamine on the battlefield and at field hospitals to determine that the dose of ketamine was not significantly associated with the development of ASD. (*P* = 0.420)

## CONCLUSIONS

In summary, the results of both the 2005 and 2008 *Schönenberg et al.* studies suggested that peritraumatic ketamine worsened symptoms of ASD and led to elevated PDEQ scores. However, the later-conducted studies by *Usuki et al. (2012)* and *Mion et al. (2017)*, found that ketamine had no effect on the development of PTSD. Although the ISS subject scores were comparable across the four study samples, it remains unclear how the number/types of surgical procedures could have influenced the experiences of patients in these different studies.

Based on the studies to date, the factors contributing to onset and prevention of trauma related ASD and PTSD appear to be complex and the literature to date pertaining to potential agents of prevention (e.g., ketamine) is both sparse and discrepant. Contemporary ED and ICU clinicians who provide trauma care should be cautious in their use of ketamine for this therapeutic purpose.

Primary care and psychiatry clinicians may also still see ASD and PTSD patients who have received these prescribed medications during subsequent office visits.

In the future, larger clinical controlled studies regarding the use of peritraumatic ketamine and other medications for the management of ASD and PTSD will certainly be required.

### Conflict of Interest

The authors declare no conflicts of interest.
